# An Inguinal Perivascular Epithelioid Cell Tumor Metastatic to the Orbit

**DOI:** 10.1155/2018/5749421

**Published:** 2018-05-29

**Authors:** Zofia Tynski, Way Chiang, Albert Barrett

**Affiliations:** ^1^Department of Pathology, Clarion Hospital, Clarion, PA 16214, USA; ^2^Department of Family Medicine, Clarion Hospital, Clarion, PA 16214, USA; ^3^Department of Radiology, Clarion Hospital, Clarion, PA 16214, USA

## Abstract

Malignant PEComas are rare mesenchymal neoplasms. These tumors harbor distinct myomelanocytic phenotype. The PEComa family of tumors includes lymphangioleiomyomatosis, angiomyolipoma, clear cell sugar tumor of the lung, and myomelanocytic tumor of the falciparum ligament/ligamentum teres. PEComas have no known normal cell counterpart. Majority of PEComas are benign and occur predominantly in the middle-age women. These tumors are commonly encountered in the uterus. Herein, we report a 20-year-old woman with a left inguinal mass metastatic to orbit, brain, lumbar spine, and skin at presentation. To our knowledge, this is the first case of metastatic PEComa to the orbit. This is the third case of primary PEComa of the inguinal area.

## 1. Introduction

Bonnetti et al. first noted in 1992 an unusual cell lineage that expressed both melanocytic and smooth muscle markers with an apparent epithelioid morphology, giving rise to perivascular epithelioid cells (PECs) [1]. The hallmark of PECs is their characteristic perivascular distribution. In 1996, Bonnetti's colleague, Zamboni, delineated perivascular epithelioid cells in both angiomyolipoma (AML) and clear cell “sugar” tumor of the lung and coined the term PEComa [2]. In 2002, World Health Organization defined PEComas as “mesenchymal tumors composed of histologically and immunohistochemically distinctive perivascular epithelioid cells” [3]. About 10% of PEComas are associated with an autosomal dominant tuberous sclerosis complex (TSC). Our patient did not have history of tuberous sclerosis.

## 2. Case Presentation

**Figure 1:**
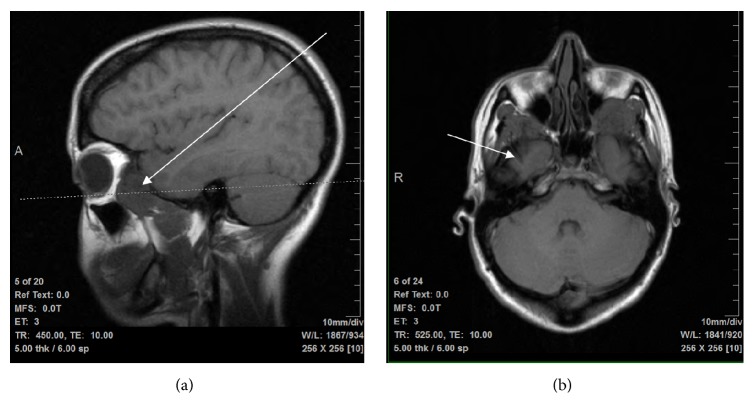
T1 MRI images of malignant PEComa metastasis exerting mass effect in both (a) sagittal and (b) axial views.

**Figure 2:**
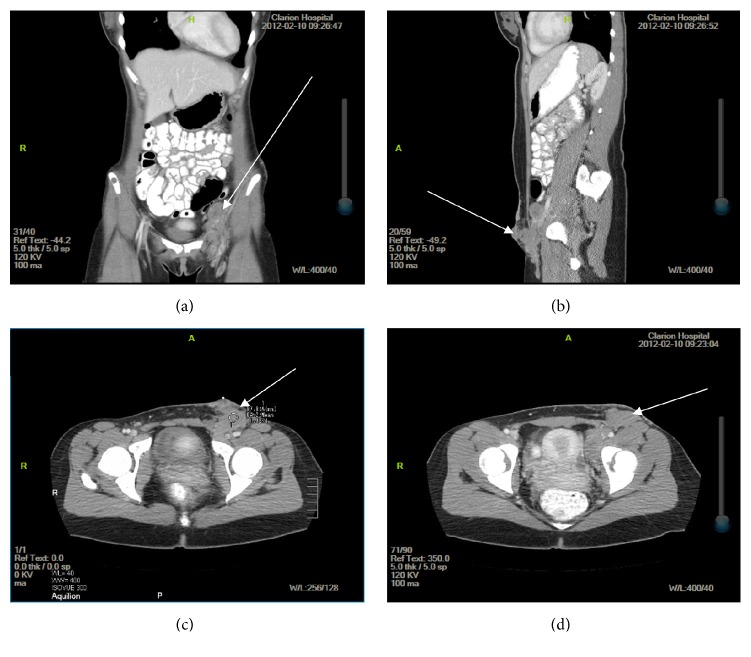
Initial CT imaging of the abdomen and pelvis with and without contrast for evaluation of the inguinal mass in (a) coronal, (b) sagittal, and (c) axial views demonstrating superficial extension and involvement of anterior abdominal wall by mass and (d) axial view demonstrating a proximal cross-sectional view of the mass that is not extending to the superficial skin.

A 20-year-old female presented to a primary care facility with a 3-month history of tender left inguinal mass. Initially, she was treated with a course of antibiotic for presumed infectious etiology. Since there was no apparent improvement, she was referred to a surgeon for an excision. The patient denied having fevers, night sweats, or weight loss. The physical exam delineated a 7.2 cm mass filling the entire inguinal canal with skin satellitosis. The mass was resected and found to be a malignant PEComa. Unfortuitously, the PET scan delineated a widely metastatic disease at presentation. The tumor was noted in the temporal lobe of the brain, orbital bones, and lumbar spine (Figures 1 and 2). The patient was placed on numerous chemotherapy regimens starting with an mTOR inhibitor, Temsirolimus. Despite various chemotherapy regimens utilized, the tumor progressed and the patient expired due to neutropenic fevers after thirty-two months since the original diagnosis.

**Figure 3:**
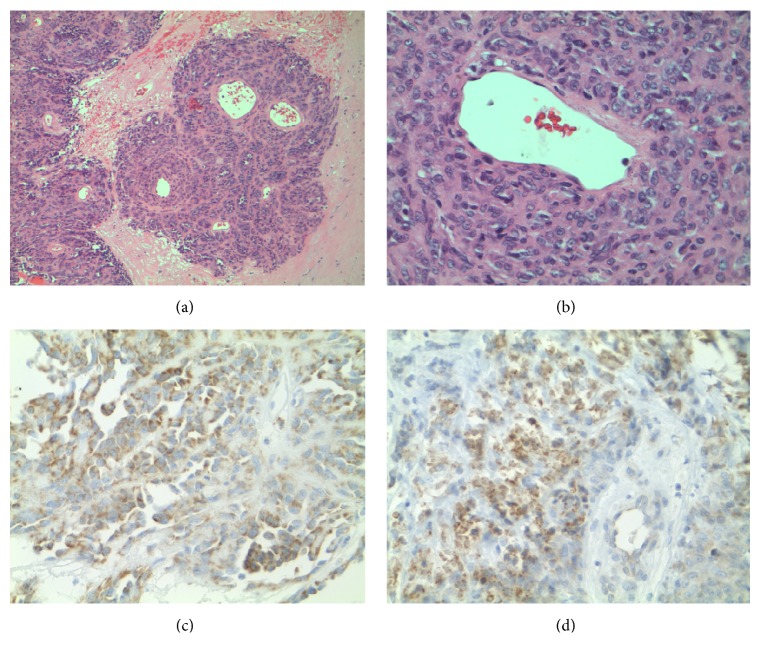
Histology and immunochemistry of malignant PEComa. (a) Malignant cells in a concentric arrangement around blood vessels (H&E, 100x). (b) Epithelioid cells with granular light eosinophilic cytoplasm and oval nuclei with small nucleoli (H&E, 400x). (c) Melan-A demonstrates salient cytoplasmic immunoreactivity, supporting melanocytic phenotype (Melan-A (MART-1), 400x). (d) HMB-45 demonstrates salient cytoplasmic immunoreactivity, supporting melanocytic phenotype (HMB-45, 400x).

**Figure 4:**
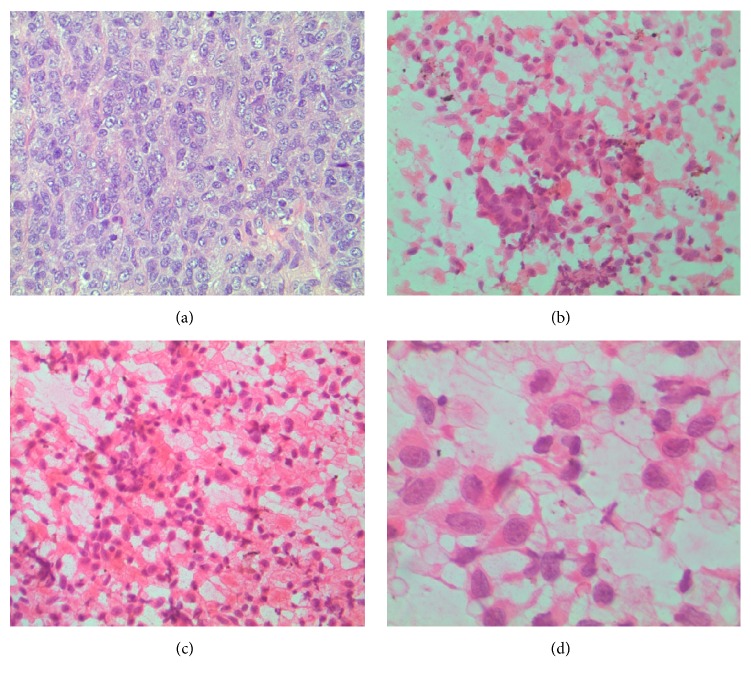
Histology and cytology of malignant PEComa. (a) Sheets of polygonal epithelial cells with hyperchromatic nuclei with prominent nucleoli and brisk mitotic activity (H&E, 400x). (b) Epithelioid cell clusters in a hemorrhagic background (Papanicolaou stain, 400x). (c) Medium cellular aspirate in hemorrhagic background (Papanicolaou stain, 400x). (d) Pleomorphic cells with enlarged nuclei with conspicuous nucleoli and light eosinophilic cytoplasm (Papanicolaou stain with oil, 1000x).

Grossly, the tumor was well defined with an ulcerated surface and it measured 7.2 cm × 2.5 cm × 2.8 cm. The cut surface was homogenous tan flesh with a prominent central hemorrhagic area. On microscopic examination, there were two patterns noted. The predominant pattern was that of the malignant cells concentrically located around hyalinized medium-sized blood vessels (Figure 3). The less prominent pattern was that of the malignant cells arranged in sheets forming cords or nodules (Figure 4). The tumor divulged conspicuous areas of hemorrhage and coagulative necrosis. The malignant cells were polygonal epithelioid cells that harbored granular eosinophilic cytoplasm with indistinct cellular borders. The cells demonstrated variably round to oval nuclei. The nuclei were equipped with vesicular chromatin or prominent nucleoli. The mitotic index was brisk (>20 per HPF). Immunohistochemically, the tumor cells were positive for HMB45, Melan A, MiTF, vimentin, and ERG but negative for CD31, CD34, p63, EMA, CAM 5.2, CK7, AE1/3, S100, SMA, desmin, and TFE3.

The cytology delineated an epithelioid cellular aspirate in a hemorrhagic background. The cells had hyperchromatic nuclei with an amphophilic cytoplasm. The nuclei had conspicuous nucleoli and some binucleated cells were appreciated. Occasionally, intranuclear inclusions were noted (Figure 4).

## 3. Discussion

To our knowledge, this is the first case of malignant PEComa metastatic to orbit and only the third case of primary inguinal PEComa. Currently, there are five cases of primary orbital PEComas reported in English literature. Since PEComas occur in females, gynecological sites predominate. It is believed that 40% occur in uteri [4]. The median age at presentation is 43 years [5]. However, the tumors exhibit great variability as to location and can essentially be found in such sites as skin, nasopharynx, heart, breast, bones, gastrointestinal, genitourinary tract, and brain [5].

Our case had biphasic morphology. The predominant pattern was nested with a solid epithelioid component. The epithelioid cells stained for melanocytic markers but did not stain with a myogenic marker such as SMA (Smooth Muscle Actin). In 2005, Folpe et al. demonstrated that most sensitive immunohistochemical stain for a PEComa is HMB45 (92%), followed by Melan-A (72%) and MiTF (50%) [6, 7]. In addition, the myogenic markers that are immunoreactive include SMA (80%), desmin (36%), S100 (33%), vimentin (86%), pan-cytokeratin (13%), TFE3 (29%), and CD117 (5%) [6, 7]. Our case delineated melanocytic immunoreactivity for HMB45, Melan-A, and MiTF but it failed to express SMA or desmin. It appears that immunohistochemical reactivity of PEComa is inherently linked to morphology. Furthermore, the epithelioid predominant PEComas have a strong predilection for melanocytic markers, while the spindle predominant PEComas stain strongly with myogenic markers [8].

WHO defined PEComas as a mesenchymal tumor for the first time in 2002. The PEComa cells have three hypotheses of origin. One is that they are derived from neural crest cells. Another hypothesis is that they are myoblastic (smooth muscle) in nature. Others postulate that these cells originated from pericytes, which are essentially progenitors of fat/muscle cells [9, 10].

PEComas confer to a three-tier classification system designed by Dr. Folpe et al. ([6], Table 1). The three diagnostic categories are the following: benign, uncertain malignant potential, and malignant. This classification identifies six high risk features. These are size > 5 cm, an infiltrative growth pattern, high nuclear grade and cellularity, mitotic rate > 1/50 HPF, necrosis, and vascular invasion. Benign PEComas are devoid of all high risk features. The PEComas of uncertain malignant potential can harbor one of the following: nuclear pleomorphism and multinucleated giant cell or being greater than 5 cm in size. The malignant category is reserved for PEComas that demonstrate 2 or more high risk features.

There are few entities to consider in the differential diagnosis of a malignant PEComa. Melanoma and PEComa both stain with specific melanocytic markers such as HMB45, Melan-A, and MiTF. However, only melanoma delineates robust and diffuse staining with a sensitive melanocytic marker such as S-100. Notably, PEComa is known to be immunoreactive in 11% of cases [11]. This tumor lacked completely any immunoreactivity for S-100 antibody. Both melanoma and PEComa can express vimentin, though melanoma does not express other myogenic markers. A conspicuous concentric perivascular arrangement seen in a PEComa is not a compelling feature of melanoma, which tends to show a more discohesive pattern. Lastly, our patient did not have an apparent melanoma in situ component, which is usually inherently present in a melanoma. Most of the tumor burden was contained in deep mesenchymal tissue rather than skin. Furthermore, clear cell sarcoma (melanoma of soft parts) also stains strongly and diffusely with S-100 but PEComa does it infrequently. In addition, the hallmark for diagnosing clear cell sarcoma is the signature translocation, *t*(12; 22), with the fusion of EWS-ATF1 gene. Gastrointestinal stromal tumor can be epithelioid in morphology but does not stain for melanocytic markers. The GIST tumor stains for DOG1, CD34, and CD117. The latter can be positive in 5% of PEComas. An angiosarcoma is also in the differential. The majority of these tumors are associated with postradiotherapy and c-myc can be apparent in these cases [12, 13]. An angiosarcoma may stain for CD31, CD34, ERG, and SMA but not with melanocytic markers. Lastly, an alveolar soft part sarcoma (ASPS) can have similar morphology to a PEComa, but lack of melanocytic expression readily distinguishes the two. They both express myogenic immunoreactivity. Furthermore, TFE3 delineates nuclear positivity in ASPS due to an unbalanced translocation in der(17)*t*(x; 17) (p11.2; q25), which yields the TFE3-ASPL gene product. The TFE3 immunoreactivity is readily reported in 29% of PEComas with prominent epithelioid morphology.

The treatment of choice for PEComas is surgical resection. Malignant PEComas are not chemo- or radiosensitive [10]. In metastatic disease, PEComas are treated with mTOR inhibitors. Through the genetic association of tuberous sclerosis, which happens to be in 10% of the time, loss of tuberous sclerosis complex gene is known (TSC1/TSC2). These genes are tumor suppressor genes that can destabilize the rapamycin (mTOR) pathway [14]. In tuberous sclerosis patients, there is one normal copy of either TSC1 or TSC2 gene; then another aberrant genetic process takes place in a form of mutation, deletion, or partial inactivation of an aberrant second gene copy, which instigates uninhabited activation of mTOR pathway and subsequently uncontrolled tumor production [15]. Since many patients do not respond to mTOR inhibitors like our patient, it is postulated that malignant PEComas exhibit molecular variability.

In conclusion, we present a first case of metastatic malignant PEComa to the orbit in a 20-year-old female. The patient had undergone surgical resection and was found to be metastatic at presentation. She was treated very aggressively with multiple chemotherapeutic agents including mTOR inhibitors. After thirty-two months of protracted disease course, the patient succumbed to a neutropenic fever.

## Figures and Tables

**Table 1:** Classification of PEComas (adapted from Bleeker and Folpe, [5, 6]).

High risk features
Size > 5 cm
Infiltrative growth pattern
High nuclear grade and cellularity
Mitotic rate > 1/50 HPF
Necrosis
Vascular invasion
Benign
No high risk features
Uncertain malignant potential
1 high risk feature including size ≥ 5 cm, nuclear pleomorphism, or multinucleated giant cell
Malignant
2 or more high risk features
